# Particulate Matter Exposure after a Cancer Diagnosis and All-Cause Mortality in a Regional Cancer Registry-Based Cohort in South Korea

**DOI:** 10.3390/ijerph19169875

**Published:** 2022-08-10

**Authors:** Sang-Yong Eom, Yong-Dae Kim, Heon Kim

**Affiliations:** 1 Department of Preventive Medicine, College of Medicine, Chungbuk National University, Cheongju 28644, Korea; 2 Chungbuk Environmental Health Center, Chungbuk National University Hospital, Cheongju 28644, Korea; 3 Chungbuk Regional Cancer Center, Chungbuk National University Hospital, Cheongju 28644, Korea

**Keywords:** particulate matter, cancer, patient survival, all-cause mortality

## Abstract

Although particulate matter (PM) is a Group 1 carcinogen, few studies have evaluated the effect of PM exposure after a cancer diagnosis on survival. Herein, we evaluated the effect of exposure to ambient PM_10_ after a cancer diagnosis on survival using data from the Regional Cancer Registry cohort in Chungbuk Province, Korea. A total of 44,432 patients with cancer who survived for >1 year after being diagnosed between 2005 and 2018 were followed until 31 December 2019; there were 32,734 survivors (73.7%) and 11,698 deceased (26.3%). The average follow-up period was 67.7 months, and the cumulative average concentration of PM_10_ exposure of patients with cancer after a diagnosis was 49.0 µg/m^3^. When PM_10_ concentration increased by 1 standard deviation (5.2 µg/m^3^), the all-cause mortality risk increased 2.06-fold (95% CI: 2.02–2.11). This trend was most pronounced in the younger patient group and in patients with local-stage cancer. This study demonstrates that exposure to PM_10_ after cancer diagnosis might influence the survival of patients with cancer, requiring environmental preventive measures such as lower pollutant exposure.

## 1. Introduction

It is estimated that there were approximately 19.3 million cases of cancer and 10 million cancer deaths worldwide in 2020 [1]. In 2019, the number of new cases of cancer in Korea was 254,718, with 1.14 out of 3 Koreans expected to develop cancer in their lifetime [2]. The 5-year relative survival rate of Korean patients with cancer has drastically increased from 41.2% in 1995 to 70.3% in 2014 due to recent improvements in treatment modalities and the introduction of comprehensive cancer management programs. However, the improvement in the 5-year relative survival rate has slowed since 2014, stagnating at 70.7% in 2019 [2]. This highlights the necessity to identify factors related to early deaths of patients and to develop additional interventions. Some studies have suggested the need for research to study the effects of air pollution exposure on the survival of patients with cancer after a diagnosis [3,4].

The International Agency for Research on Cancer classifies particulate matter (PM) as a Group 1 carcinogen in humans [5]. PM causes cancer through DNA damage caused by electrophilic compounds and gene expression changes mediated via epigenetic alterations [4,6]. PM exposure triggers reactive oxygen species production, leading to oxidative stress and subsequent inflammatory responses, which may become chronic under prolonged exposure [6,7]. Taken together, PM exposure can promote cancer initiation and progression [4].

Following diagnosis, patients with cancer adjust their lifestyle by implementing healthy lifestyle adaptations, such as smoking cessation, abstinence from alcohol, diet improvement and physical exercise [8]. However, relatively little attention has been paid to interventions in the residential environment of patients with cancer that are directly related to air pollutant exposure. PM exposure following cancer development may represent an important determinant of patient survival, with several recent studies having reported a relationship between PM_10_ exposure and survival [9,10,11]. However, most of these studies were conducted in countries with relatively low levels of PM_10_. Only a few studies have focused on the impact of PM exposure on cancer survival after diagnosis in the East Asia region, where atmospheric PM_10_ concentrations are relatively high. Thus, in the present study, we aimed to evaluate the association between PM_10_ exposure and cancer survival rate after a diagnosis through the analysis of Regional Cancer Registry data from North Chungcheong Province (i.e., Chungbuk), Korea. We determined that all-cause mortality was indeed related to PM exposure.

## 2. Materials and Methods

### 2.1. Study Population

The retrospective cohort, based on Chungbuk Regional Cancer Registry (CBRCR) data, consisted of a total of 94,720 patients with cancer in Chungbuk Province, Korea, from 1 January 2005 to 31 December 2018. The data, which were provided by Chungbuk Regional Cancer Center, included age at the time of cancer registration, sex, address (e.g., city, county and district), cancer diagnosis ICD-10 code, cancer stage, death status and date of death. This study was reviewed and approved by the Institutional Review Board at Chungbuk National University and was exempted from obtaining the written consent of subjects, as we used secondary data without personally identifiable information (CBNU-202107-0112).

### 2.2. PM Exposure

The PM_10_ exposure data were obtained from the National Air Quality Monitoring Information Network of the Ministry of Environment. There are 18 stations in 11 administrative districts in Chungbuk, and the PM_10_ concentration was measured using the beta-ray absorption method [12] (Supplementary Figure S1). We collected data on the monthly averages of PM_10_ concentrations from 2005 to 2019 at all air pollution monitoring stations in Chungbuk province. Individual exposure to PM_10_ after a cancer diagnosis was calculated based on the data from the monitoring stations near the residences of patients with cancer and by calculating the cumulative average exposure concentrations from the time of cancer registration to the time of final follow-up (31 December 2019 or the date of death).

### 2.3. Cohort Database Construction

**Figure 1:**
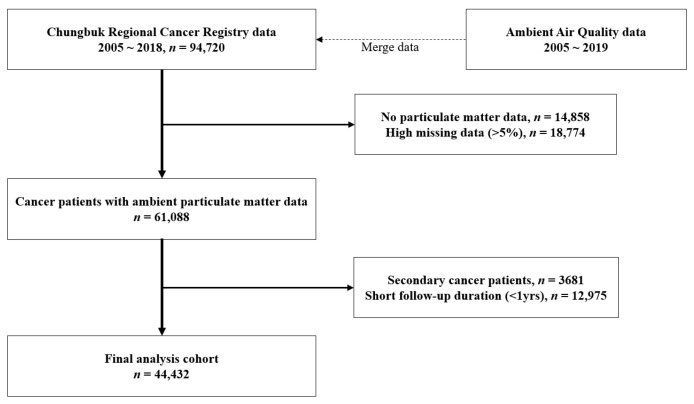
Flowchart of study cohort selection.

CBRCR data were merged with the data on individual PM_10_ exposure. The cases in which no data on PM_10_ exposure were available (*n* = 14,858) or in which the missing rate of PM_10_ exposure data was more than 5% (*n* = 18,774) were excluded. Furthermore, the final analysis excluded the data of patients with a follow-up period of less than 12 months (*n* = 12,975) and those with secondary cancer (*n* = 3681) that was different from the first in order to evaluate the effects of chronic exposure to air pollutants. A total of 44,432 patients were finally selected for analysis (Figure 1). The start time of cohort follow-up was based on the cancer registration date, and the end date was set as 31 December 2019 or the date of death. As the CBRCR data did not contain information on potential confounders, such as individuals’ smoking, drinking and socioeconomic level, the information was collected from the regional data of the Korean Statistical Information Service (KOSIS).

### 2.4. Statistical Analysis

The hazard ratios (HRs) and 95% confidence intervals (CIs) were calculated for all-cause mortality in relation to PM_10_ exposure after cancer diagnosis by using the Cox proportional hazards model. Model 1 encompassed age and sex as covariates, while Model 2 also included the summary stage of cancer, smoking, drinking and socioeconomic levels as covariates. We evaluated the proportional hazard assumption of Cox models, which showed that none of the predictors violated the proportional hazards assumption. Stratified analysis was performed based on age, gender, cancer stage at the time of diagnosis and follow-up period. The significance level was set to *p* < 0.05, and statistical analysis was performed using SPSS Statistics Version 25.0 (IBM Corp., Armonk, NY, USA).

## 3. Results

Table 1 shows general characteristics of the study cohort. The average age of 44,432 patients with cancer at the time of diagnosis was 58.1 years, and males accounted for 49.5% of the cohort. At the time of diagnosis, the most common stage was “localized” in 48.0% of the cohort, followed by “regional” in 33.0% of the cohort, “distant” in 11.3% of the cohort and “unknown” in 7.8% of the cohort. The average follow-up period was 67.7 months, and the average concentration of PM_10_ exposure after a cancer diagnosis was 49.0 µg/m^3^.

Following diagnosis, the all-cause mortality risk increased by 2.06-fold (95% CI: 2.02–2.11) when the average PM_10_ concentration increased by 1 standard deviation (5.2 µg/m^3^). The group with a PM_10_ exposure concentration of 60 µg/m^3^ or higher exhibited 9.67-fold (95% CI: 9.08–10.29) higher all-cause mortality compared to the group exposed to 50 µg/m^3^ or lower (Table 2).

The mortality risk from PM_10_ exposure after cancer diagnosis was highest in the age group of <15 years old (HR: 4.77, 95% CI: 3.37–6.76), and it showed a decreasing tendency in the older age groups. The comparison by gender showed that the mortality risk was slightly higher for females than for males. With regard to cancer stage, the risk of mortality associated with PM_10_ exposure was highest at the “local” stage and became relatively lower at the “distant” and “unknown” stages. We divided the follow-up period into three segments (<30 months, 30–60 months, >60 months) to evaluate whether the association between PM_10_ exposure after a cancer diagnosis and mortality risk differed over time. Patients with a follow-up period of more than 60 months had the highest mortality risk due to increased exposure to PM_10_ after a cancer diagnosis (HR: 4.51, 95% CI: 4.16–4.89) (Table 3).

Analysis per cancer type revealed that PM_10_ exposure after diagnosis significantly increased the mortality risk of patients with all cancer types, except for patients with testicular cancer or Hodgkin’s lymphoma (Table 4). In the sensitivity analyses, the associations of exposure to PM_10_ after a cancer diagnosis with risk of all-cause mortality were consistent with the primary analysis (Supplementary Table S1).

## 4. Discussion

We analyzed a patient cohort based on Regional Cancer Registry data from the Chungbuk region, which is located in the inland of the Republic of Korea. We confirmed that PM_10_ exposure after a cancer diagnosis was associated with the all-cause mortality risk of patients with cancer. The association was clearly observed in patients with a lower age of diagnosis, as well as in those with local-stage disease. Our findings were similar to those of studies analyzing California cancer registration data, which reported that PM exposure after a diagnosis of lung cancer and hepatocellular carcinoma was related to a shorter survival period, which was mainly observed for patients with local-stage early cancer [10,13]. Furthermore, Ou et al. reported a significant association between PM_2.5_ exposure and mortality rates after cancer diagnosis in pediatric patients (aged <15 years) as well as adolescents and young adults (aged 15–39 years) [14]. Taken together, research on the matter indicates that the average cumulative PM exposure after a cancer diagnosis is an important determinant of survival in patients with cancer.

A longitudinal study of Caucasian patients with lung cancer reported that PM_10_ exposure increased cancer mortality 1.48-fold per 10 µg/m^3^ increase in exposure after adjusting for demographic factors and cancer characteristics [13]. Eckel et al. reported that the HR for all-cause mortality associated with an increase of 1 SD (12.1 µg/m^3^) in PM_10_ was 1.11 (95% CI: 1.11–1.12) in patients with lung cancer after adjusting for potential confounders (including stage and histology) [9]. A recent pan-cancer study reported a minimal association of PM_2.5_ with all-cause mortality (HR: 1.01, 95% CI: 1.00–1.03) per 10 µg/m^3^ increase in PM_2.5_ [11]. The current findings cannot be directly compared with these previous results, as we found that the risk ratios of all-cause mortality associated with an increase of 1 SD increase in PM_10_ (5.2 μg/m^3^) were 2.06 (95% CI: 2.02–2.11) and 1.46 (95% CI: 1.40–1.53) in patients with any cancer type and lung cancer, respectively. The results may depend on differences in PM_10_ exposure sources and concentration levels, as well as on the demographic characteristics of the target group. In addition, it suggests that exposure to PM_10_ after a cancer diagnosis may play a more important role in the mortality of patients with cancer than exposure before the onset of cancer. However, it would not be possible to completely separate the effects of exposure to PM_10_ before and after a cancer diagnosis on the mortality of cancer patients. The association observed in this study between PM exposure after diagnosis and mortality should be approached cautiously.

Previous epidemiological studies have evaluated the relationship between PM exposure and mortality per cancer type in Korean patients. Hwang et al. indicated that breast cancer mortality significantly increased by approximately 5% when PM_10_ increased by 10 μg/m^3^ [15]. Kim et al. estimated that the annual number of premature deaths from lung cancer caused by PM_2.5_ in Korea was approximately 5000 [16]. Shin et al. analyzed a cohort from the Seoul metropolitan area and did not find any connection between PM_2.5_ and cancer mortality. However, the HRs of PM_2.5_ were relatively higher for lung, stomach, pancreas, non-Hodgkin’s lymphoma, prostate, esophagus, oral, pharynx and brain cancer mortality (HRs: 1.44–7.14) [17]. The study by Shin et al. differs from the present study in terms of target groups and the duration of PM exposure. Shin et al. analyzed the general population in the Seoul metropolitan area, whereas the current study included residents from mid-urban and rural areas with populations of less than 1 million people. Furthermore, Shin et al. set the period of PM_2.5_ exposure to 5 years before cohort entry, whereas we set the period of PM_10_ exposure from the time of cancer diagnosis to the occurrence of an event or the end of follow-ups.

The average follow-up period of patients with cancer in the present study was 68 months, and the average cumulative PM_10_ concentration during the study period was 49.0 μg/m^3^, which is 2.5-fold higher than the WHO recommendation standard (20.0 μg/m^3^). The study site, Chungbuk, has the geographical feature of an inland basin located in the center of Korea with various PM emission facilities, such as industrial complexes, incineration facilities and cement factories. Our previous studies found that living near environmentally hazardous facilities in the Chungbuk region increased the risk of cancer [18,19,20]. These results suggest the need for environmental interventions in such residential areas in order to lower cancer incidence and mortality. A quasi-experimental study on the effect of air quality management policies in the metropolitan area confirmed that environmental interventions were effective in reducing cardiovascular mortality [21]. Our previous study also confirmed that an air purifier contributed to lowering indoor PM exposure and oxidative stress in patients with cardiovascular disease [22].

The biological mechanism underlying the association between PM exposure and survival of patients with cancer remains unclear. It is expected that the oxidative stress and systemic inflammatory response caused by PM exposure may affect disease progression [4,5,6,7]. PM-induced oxidative stress activates inflammatory pathways and can influence tumor cell survival, proliferation, chemical resistance, radiation resistance, invasion and angiogenesis [23]. Fruit and vegetable consumption after cancer diagnosis has been reported to reduce cancer mortality, highlighting the importance of oxidative stress control after diagnosis [24,25]. In the same context, the use of aspirin and other non-steroidal anti-inflammatory drugs was associated with a higher cancer survival rate, indicating the importance of suppressing inflammation after a cancer diagnosis [26,27].

The current study has a few limitations. First, the assessment of individual PM_10_ exposure may be inaccurate, as area-level concentration data obtained from the measurement network of fixed monitoring stations near patient residences were used as a proxy for individual exposure to PM_10_. The reliability of exposure estimates can be significantly affected by the spatial distribution and density of local monitors. Area-level exposure estimates based on patient residences may not fully reflect the individual exposure level because the actual exposure varies according to individual activity patterns and time spent indoors. Furthermore, the information on relocation or long-term hospitalization at other local medical institutions after a cancer diagnosis was not accounted for in the analysis. Nevertheless, this area-level approach to exposure assessment is a realistic surrogate indicator when direct individual assessment is not impossible. In addition, it was assumed that there were random errors in measuring the amount of PM_10_ exposure, which probably led to the null result rather than an overestimation of the association between PM exposure and mortality. Second, most previous studies on the relationship between PM exposure and cancer survival focused on PM_2.5_. We used PM_10_ data, as PM_2.5_ measurements were not available. Therefore, the results of this study cannot be directly compared with those of research that focused on PM_2.5_. Third, this study was based on regional cancer registry data, which did not include information on potential confounders at the individual level (e.g., lifestyle habits such as smoking, drinking, diet, occupation, socioeconomic level and comorbidities). However, it is unlikely that such variables are directly related to atmospheric PM exposure, which is the explanatory variable in our study. In the final model, we adjusted for regional-level data on smoking and income. Finally, as this study was based on the cancer registration data of a single region in Korea, additional studies should extend the current results. 

## 5. Conclusions

This study confirmed that PM exposure after a cancer diagnosis is associated with lower patient survival. The current findings highlight the need for corresponding preventive measures, such as lowering pollutants exposure.

## Figures and Tables

**Table 1:** General characteristics of the study cohort.

	Total
Number, *n* (%)	44,432 (100.0)
Age at diagnosis (year), mean (SD)	58.1 (14.9)
Sex, *n* (%)	
Men	21,980 (49.5)
Women	22,452 (50.5)
Stage at diagnosis, *n* (%)	
Local	20,428 (48.0)
Regional	14,050 (33.0)
Distant	4822 (11.3)
Unknown	3307 (7.8)
Duration of follow-up (month), mean (SD)	67.7 (45.4)
Ecological statistic ^a^, % (SD)	
Smoking status (% of smokers)	24.1 (2.1)
Drinking status (% of drinkers)	59.7 (3.1)
Gross regional domestic product	27.2 (11.3)
Ambient PM_10_ ^b^ (µg/m^3^), mean (SD)	49.0 (5.2)

^a^ The ecological statistic is a representative value for the patient’s residential area level. ^b^ PM_10_ mean concentration during follow-up period. SD: standard deviation, PM: particulate matter.

**Table 2:** HRs for all-cause mortality by continuous and categorized PM_10_ exposure.

	HR (95% CI)	
	Model 1	Model 2
Continuous PM_10_ (one SD increase)	1.79 (1.76–1.82)	2.06 (2.02–2.11)
Categorized PM_10_		
<50 µg/m^3^	1.00 (ref.)	1.00 (ref.)
50–60 µg/m^3^	1.59 (1.53–1.66)	2.05 (1.96–2.14)
≥60 µg/m^3^	7.88 (7.47–8.32)	9.67 (9.08–10.29)

Model 1 adjusted for age and sex. Model 2 further adjusted for cancer stage (SEER code) and ecological covariates (smoking, drinking and regional gross in area level). HR: hazards ratio, CI: confidence interval, SD: standard deviation, PM: particulate matter.

**Table 3:** HRs for all-cause mortality with an increase of 1 standard deviation in PM_10_ exposure, stratified by age, stage at diagnosis and sex.

		HR (95% CI)	
		Model 1	Model 2
Age at diagnosis (year)	<15	3.33 (2.45–4.52)	4.77 (3.37–6.76)
	15–34	2.88 (2.48–3.34)	3.72 (3.17–4.37)
	35–64	2.22 (2.16–2.29)	2.53 (2.45–2.61)
	≥65	1.55 (1.51–1.58)	1.75 (1.70–1.79)
Sex	Men	1.71 (1.67–1.75)	1.96 (1.92–2.02)
	Women	1.87 (1.82–1.93)	2.11 (2.04–2.18)
Stage at diagnosis	Local	2.00 (1.93–2.08)	2.49 (2.39–2.60)
	Regional	1.78 (1.72–1.84)	2.15 (2.07–2.23)
	Distant	1.34 (1.30–1.38)	1.51 (1.46–1.56)
	Unknown	1.56 (1.49–1.65)	1.78 (1.69–1.89)
Follow-up duration	<30 months	1.49 (1.46–1.51)	1.36 (1.33–1.39)
	30–60 months	1.90 (1.86–1.95)	1.59 (1.54–1.65)
	>60 months	2.48 (2.34–2.63)	4.51 (4.16–4.89)

Model 1 adjusted for age and sex. Model 2 further adjusted for cancer stage (SEER code) and ecological covariates (smoking, drinking and regional gross in area level). HR: hazards ratio, CI: confidence interval.

**Table 4:** HRs for all-cause mortality by an increase of 1 standard deviation in PM_10_ exposure stratified by cancer type.

		HR (95% CI)	
	*n*	Model 1	Model 2
Lip, oral cavity and pharynx	558	1.73 (1.50–1.98)	2.08 (1.77–2.44)
Esophagus	339	1.51 (1.32–1.72)	1.58 (1.36–1.85)
Stomach	7622	2.11 (2.02–2.21)	2.25 (2.14–2.37)
Colon and rectum	6679	1.80 (1.72–1.88)	1.98 (1.89–2.08)
Liver	2023	1.52 (1.44–1.61)	1.71 (1.60–1.82)
Gallbladder	703	1.28 (1.17–1.39)	1.44 (1.30–1.60)
Pancreas	470	1.26 (1.16–1.37)	1.33 (1.22–1.46)
Larynx	265	1.50 (1.22–1.86)	1.74 (1.34–2.25)
Lung	3008	1.39 (1.33–1.44)	1.46 (1.40–1.53)
Breast	4646	2.59 (2.34–2.88)	3.01 (2.67–3.40)
Cervix uteri	1049	2.07 (1.77–2.41)	2.63 (2.20–3.14)
Corpus uteri	545	2.11 (1.67–2.65)	2.50 (1.92–3.26)
Ovary	523	1.41 (1.22–1.64)	1.48 (1.25–1.75)
Prostate	2255	1.66 (1.52–1.80)	2.06 (1.86–2.28)
Testis	69	2.07 (0.54–8.02)	2.12 (0.63–7.13)
Kidney	918	1.88 (1.64–2.17)	2.19 (1.84–2.59)
Bladder	907	1.60 (1.43–1.79)	1.85 (1.61–2.12)
Brain and central nervous system	362	1.42 (1.23–1.63)	1.57 (1.34–1.84)
Thyroid	6770	2.86 (2.28–3.59)	5.83 (4.40–7.74)
Hodgkin lymphoma	45	2.84 (1.00–8.08)	4.10 (0.77–21.78)
Non-Hodgkin lymphoma	742	1.47 (1.28–1.69)	1.86 (1.59–2.18)
Multiple myeloma	244	1.56 (1.35–1.80)	1.84 (1.54–2.19)
Leukemia	584	1.92 (1.66–2.22)	2.48 (2.11–2.93)
Other and ill-defined	3106	1.71 (1.60–1.83)	1.97 (1.83–2.13)

Model 1 adjusted for age and sex. Model 2 further adjusted for cancer stage (SEER code) and ecological covariates (smoking, drinking and regional gross in area level). HR: hazards ratio, CI: confidence interval.

## Data Availability

Not applicable.

## Supplementary Materials

The following supporting information can be downloaded at: www.mdpi.com/article/10.3390/ijerph19169875/s1, Table S1. Sensitivity analyses: hazard ratios for all-cause mortality by an increase of 1 standard deviation in PM10 exposure after a cancer diagnosis. Figure S1. Location of air pollution monitoring station at each district in Chungbuk Province in Korea.
