# Supplementary material for: Particulate Matter Exposure after a Cancer Diagnosis and All-Cause Mortality in a Regional Cancer Registry-Based Cohort in South Korea
Source: Int J Environ Res Public Health. 2022 Aug 10;19(16):9875. doi: 10.3390/ijerph19169875 (PMC9408397; doi:10.3390/ijerph19169875)
Supplement: Supplementary file 1 [file ijerph-19-09875-s001.zip › ijerph-1829233-supplementary.pdf]

Table S1. Sensitivity analyses: hazard ratios for all-cause mortality by an increase of 1 standard deviation in PM_{10} exposure after a cancer diagnosis.

Sensitivity	HR (95% CI)
Inclusion of patients with cancer who had missing rates for PM_{10} data more than 5%	1.78 (1.75–1.81)
Inclusion of patients with cancer who had a short follow-up duration (< 1 years)	1.71 (1.69–1.74)
Exclusion of patients with cancer who survived less than 3 years	2.19 (2.11–2.28)
Exclusion of patients with cancer who survived less than 5 years	2.55 (2.39–2.72)
Exclusion of patients with cancer who survived less than 7 years	2.39 (2.15–2.66)
Cancer cohort consisting of patients who were diagnosed between 2005–2011	3.22 (3.12–3.33)
Cancer cohort consisting of patients who were diagnosed between 2012–2018	2.16 (2.06–2.25)

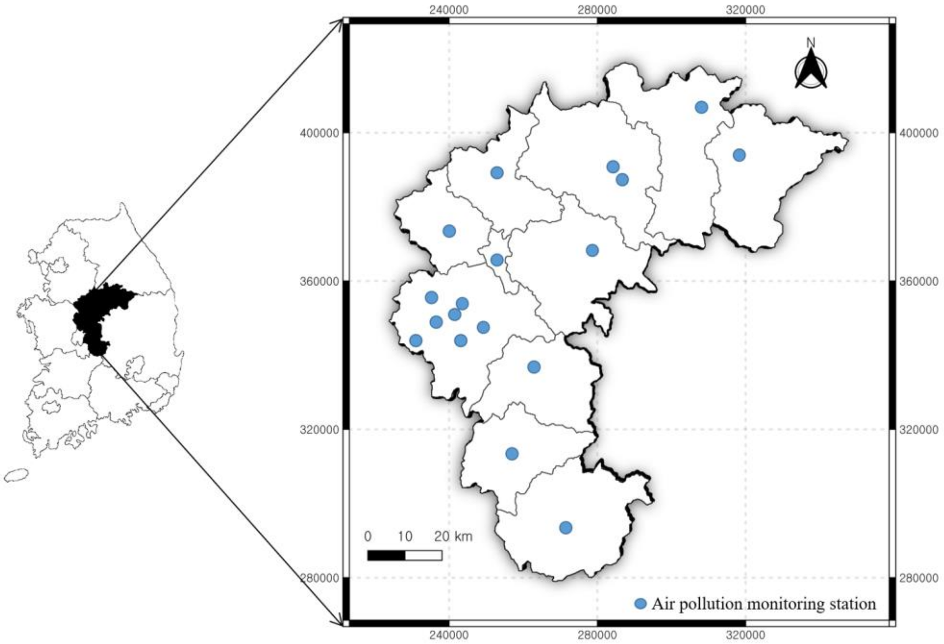

Figure S1. Location of air pollution monitoring station at each district in Chungbuk Province in Korea.
